# Phylodynamic Inference with Kernel ABC and Its Application to HIV Epidemiology

**DOI:** 10.1093/molbev/msv123

**Published:** 2015-05-29

**Authors:** Art F.Y. Poon

**Affiliations:** ^1^BC Centre for Excellence in HIV/AIDS, Vancouver, BC, Canada; ^2^Department of Medicine, University of British Columbia, Vancouver, BC, Canada; ^3^Faculty of Health Sciences, Simon Fraser University, Burnaby, BC, Canada

**Keywords:** phylodynamics, approximate Bayesian computation, tree shape, molecular epidemiology, virus evolution, human immunodeficiency virus

## Abstract

The shapes of phylogenetic trees relating virus populations are determined by the adaptation of viruses within each host, and by the transmission of viruses among hosts. Phylodynamic inference attempts to reverse this flow of information, estimating parameters of these processes from the shape of a virus phylogeny reconstructed from a sample of genetic sequences from the epidemic. A key challenge to phylodynamic inference is quantifying the similarity between two trees in an efficient and comprehensive way. In this study, I demonstrate that a new distance measure, based on a subset tree kernel function from computational linguistics, confers a significant improvement over previous measures of tree shape for classifying trees generated under different epidemiological scenarios. Next, I incorporate this kernel-based distance measure into an approximate Bayesian computation (ABC) framework for phylodynamic inference. ABC bypasses the need for an analytical solution of model likelihood, as it only requires the ability to simulate data from the model. I validate this “kernel-ABC” method for phylodynamic inference by estimating parameters from data simulated under a simple epidemiological model. Results indicate that kernel-ABC attained greater accuracy for parameters associated with virus transmission than leading software on the same data sets. Finally, I apply the kernel-ABC framework to study a recent outbreak of a recombinant HIV subtype in China. Kernel-ABC provides a versatile framework for phylodynamic inference because it can fit a broader range of models than methods that rely on the computation of exact likelihoods.

## Introduction

Phylodynamics is an emerging field in the study of RNA viruses with the central premise that the shape of a phylogenetic tree relating different infections is structured by the immunological and epidemiological environments of the virus (Grenfell et al. 2004). A phylogenetic tree is a hypothesis about how populations are related by common ancestors. Each branch in the tree represents the amount of evolutionary time that has passed since a population has diverged away from its common ancestor with another sampled population. Varying rates of lineage extinction driven in part by the host environment experienced by each population, such as the immune response, can determine which populations persist long enough to be sampled. Furthermore, the birth of new lineages is shaped by transmission of the virus between hosts, although the virus phylogeny is not necessarily concordant with its transmission history due to incomplete lineage sorting (Pamilo and Nei 1988). Under the influence of these processes that operate within and among hosts, phylogenetic trees reconstructed from genetic sequences tend to exhibit shapes characteristic of that virus species (Grenfell et al. 2004). A canonical example is that trees relating avian influenza virus hemagglutinin sequences from the same serotype strongly tend toward a comb-like shape, with a high extinction rate of lineages branching from a single trunk lineage that persists between outbreaks. In contrast, trees relating human immunodeficiency virus (HIV) sequences from the same subtype are star-like in shape, due in part to an exponential accumulation of lineages with a low rate of extinction (Sharp 2002).

A key challenge in viral phylodynamics is to use tree shapes to infer the underlying characteristics of virus epidemics with greater granularity. In response, the field of phylodynamics has entered a period of explosive growth, driven in part by the development of new models and computational methods for fitting these models to viral sequence phylogenies (Volz et al. 2013). Often, these methods have been implemented in the highly popular software package BEAST (Bayesian Evolutionary Analysis by Sampling Trees; Drummond and Rambaut 2007). In this framework, the posterior probability of a tree is calculated from its prior probability—defined by either Kingman’s coalescent (Kingman 1982) or, more recently, a birth–death process (Stadler et al. 2013)—and its likelihood given a model of evolution and the observed sequence data. The inferred tree becomes a nuisance variable over which one must integrate, given that the goal is to estimate the posterior distribution of critical parameters in an epidemic model, such as the basic reproduction number (*R*_0_). Additionally, various smoothing strategies can be employed to reconstruct dynamics in these parameters over time (Drummond et al. 2005; Stadler et al. 2013). The Bayesian approach confers robustness to uncertainty in reconstructing the tree relating observed sequences. As the number of sequences increases, however, the space of all possible trees expands at a superexponential rate. In practice, attempting to analyze several hundred sequences with conventional computing hardware may require weeks to complete (unless longitudinal sampling of sequences constrains the distribution of ancestral nodes). Conversely, genetic sequences are rapidly accumulating for many RNA viruses, especially HIV and hepatitis C virus. It is not unusual for an investigator to have access to tens of thousands of sequences from a regional epidemic. In addition, we need to be able to fit more biologically realistic models to the data (Frost et al. 2014), but such models tend to involve nonlinear dynamics and become too complex for the efficient computation of exact likelihoods; in many cases there is no analytical solution for the exact likelihood. Such limitations are motivating interest in adapting more recent techniques in model inference to phylodynamics, such as approximate Bayesian computation (ABC; Ratmann et al. 2012) and particle Markov chain Monte Carlo (MCMC) (Rasmussen et al. 2014).

The basic premise of ABC is that a model can be fit to the observed data by using the model to simulate more data sets under varying parameter values, and then finding combinations of model parameters that minimize the discrepancy between the observed and simulated data sets (Sunnåker et al. 2013). Although it can be difficult to compute the exact likelihood of a complex model, it is usually trivial to use the model to generate data simulations. The discrepancy between simulations and observations must be quantified with a distance measure. When the data are structured and complex, the distance measure may be a composite of summary measures that map the data to a more convenient metric space. For instance, the shape of a phylogenetic tree is a complex and highly structured type of data that cannot easily be reduced down to a meaningful number. However, there are a number of statistics that attempt to provide a reasonable representation of tree shape. Tree imbalance statistics, for example, quantify the degree that branching events are unevenly distributed among lineages (Mooers and Heard 1997). These statistics are intuitive and easy to compute, but they express a limited view of tree shape, do not incorporate branch lengths, and do not readily scale with tree size (but see Pompei et al. 2012). Another potential distance measure is the Robinson–Foulds metric that enumerates the number of operations required to convert one tree into another (Robinson and Foulds 1981). This metric may be more comprehensive than the imbalance statistics, but it is restricted to comparing alternative trees relating the same taxa; that is, trees with the same labels.

Kernel methods are a technique from machine learning that greatly simplify the task of comparing structured objects (Aizerman et al. 1964). In a previous study, my colleagues and I adapted a tree kernel from computational linguistics (Collins and Duffy 2002) so that it provides a distance measure for comparing phylogenetic tree shapes (Poon et al. 2013). This kernel method breaks each tree down to its constituent components (subset trees; see supplementary fig. S1, Supplementary Material online), counts the number of times each component appears in both trees, and weights this count by their concordance in branch lengths. It does not require the trees to be the same size or even to relate the same taxa. Here, I compare the performance of imbalance statistics and the kernel method for classifying trees simulated from an epidemiological model. Next, I employ the kernel method as a distance measure within the ABC framework to estimate the parameters of an epidemiological model by comparing trees generated by simulation. This approach therefore falls under the category of “kernel-ABC” methods, which is a relatively new concept in statistical inference that was first proposed in the context of population genetics (Nakagome et al. 2013). Finally, I apply this kernel-ABC method to a phylogeny generated from a recent HIV epidemic. The analyses presented here demonstrate that coupling kernel methods to ABC can accurately estimate parameters from tree shapes for a wide range of models. This is a new approach to phylodynamic inference that is highly versatile, as any model that simulates trees can potentially be fit to an observed phylogeny.

## New Approaches

To compare the shapes of different phylogenies, I have adapted a kernel function from computational linguistics that counts the number of labeled subset trees shared between two parse trees (Collins and Duffy 2002). A subset tree is a contiguous set of branches that descend from a given internal node in the tree; thus, it does not necessarily contain all of the descendants of that node (supplementary fig. S1, Supplementary Material online). To compute the kernel function, subset trees in two phylogenies are matched by topology as defined by the configuration of internal and terminal nodes. Phylogenies were “ladderized” to maximize their potential for overlapping topologies. Matching subset trees were penalized by their overall size and by their discordance in branch lengths using a Gaussian radial basis function (Poon et al. 2013). The resulting kernel function provides a comprehensive similarity measure for phylogenetic tree shapes. Thus, it is a potentially useful measure to fit models to phylogenies by ABC (Sunnåker et al. 2013), where model parameter values are evaluated by simulating data sets and comparing them to the observed data through one or more similarity measures. The best-fitting parameter values should yield simulations that most closely resemble the observed data. I implemented a class of ABC procedures known as ABC–MCMC, in which simulation-based inference is carried out within a MCMC framework (Marjoram et al. 2003). Proposals were generated by drawing a new parameter assignment from a multivariate distribution centered at the current parameter values. Instead of taking the conventional approach of rejecting proposals at a distance from the observed data beyond some cutoff value, I used an exponential weighting kernel with simulated annealing to improve convergence properties of the ABC–MCMC process (Ratmann et al. 2012). To validate this phylodynamic “kernel-ABC” approach, I evaluated its ability to recover parameter values from trees simulated under a simple epidemic model (birth–death susceptible–infected–recovered model, BDSIR; Kühnert et al. 2014) and compared its performance to a leading software package for phylodynamic inference (BEAST2; Bouckaert et al. 2014). Furthermore, I validated the kernel-ABC approach on trees generated from a more complex epidemic model of a risk-structured population (Jacquez et al. 1988).

## Results

### Predicting Contact Rates

**Fig. 1.**
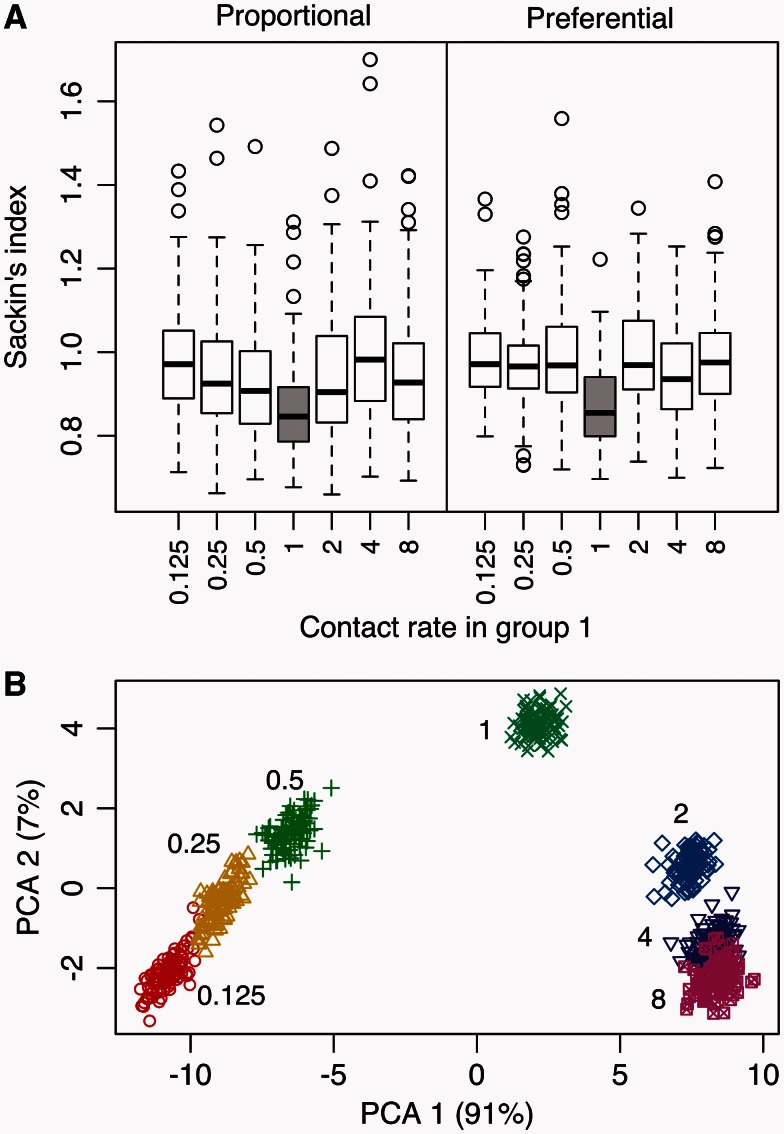
(*A*) Response of Sackin’s index (*I_S_*) to the varying heterogeneity in contact rates. Each set of box-and-whisker plots summarizes the distribution of *I_S_* for 100 replicate trees simulated under different values of *c*_1_ ($c_{1}=c_{2}=1$ is shaded for reference). Varying *c*_1_ away from $c_{2}=1$ had a more pronounced effect under preferential (ρ=0.9) than proportional (*ρ* = 0) mixing. (*B*) Projection of simulated trees to kernel space. Sets of 100 replicate trees are each annotated in the plot with their corresponding *c*_1_ values. The proportion of variation explained by the first two principal components, as estimated from the eigenvalues, is reported by the respective axis labels.

Tree imbalance statistics have been developed to detect variation in branching rates along a phylogeny (Mooers and Heard 1997). To provide the most favorable conditions for imbalance statistics, I simulated trees using a coalescent process defined by a “differential risk” epidemic model (Jacquez et al. 1988), where the host population was structured by groups with different transmission risks as determined by group-specific contact rates. Preferential transmission within groups was modeled by a mixing parameter *ρ.* Sets of 100 replicate trees with 1,000 tips each were generated for varying settings of the contact rate for individuals in one of two risk groups (*c*_1_). Varying this parameter had a pronounced effect on the shapes of the trees, as illustrated in supplementary figure S2, Supplementary Material online. Specifically, more discordant contact rates led to greater variation in branching rates, resulting in more imbalanced trees. First, I evaluated whether it was possible to differentiate among trees simulated under different values of *c*_1_ using one of several imbalance statistics. Results from using Sackin’s index (*I_S_*), which is based the average number of internal nodes separating tips from the root, are shown in figure 1*A.* Trees tended to have higher values of *I_S_* as *c*_1_ deviated in either direction away from *c*_2_, which was fixed at 1. This effect was more pronounced with preferential mixing (ρ=0.9) than when mixing was in proportion to group size (*ρ* = 0). As *I_S_* was elevated in both directions, it would be effectively impossible to determine whether contact rates in group 1 were greater or lesser than group 2. Similar outcomes were obtained using the other six imbalance statistics evaluated in this study (supplementary fig. S3, Supplementary Material online). These results are consistent with a previous numerical analysis by Frost and Volz (2013) of imbalance statistics in the context of this differential risk model.

A kernel function provides an efficient method for comparing complex objects by breaking them down into their constituent parts, and counting the number of times the same parts appear in both objects (Aizerman et al. 1964). For the tree shape kernel, these parts (features) are known as subset trees, which are contiguous sets of branches that descend from an internal node and do not necessarily include the tips (supplementary fig. S1, Supplementary Material online). Figure 1*B* displays a projection of the same simulated trees (ρ=0.9) onto the first two principal components of a pairwise similarity matrix produced by the tree shape kernel (eqs. 2–4). Taken together, these two components explained roughly 98% of the variation. This projection illustrates a clear separation of trees into distinct clusters by contact rate (*c*_1_). Similar results were obtained for trees simulated with *ρ* = 0 (supplementary fig. S4, Supplementary Material online). Unlike the imbalance statistics, the kernel was able to differentiate between trees generated under low and high values of *c*_1_ relative to *c*_2_.

To evaluate the ability of imbalance statistics to predict *c*_1_ from tree shape, simulations were restricted to trees generated with either $c_{1}\leq 1$ or $c_{1}\geq 1$. Under these conditions, the imbalance statistics were given an unfair advantage by communicating a priori that the contact rate underlying a given tree was either greater or less than 1. Under this constraint, samples of 400 trees were drawn at random and stratified by *c*_1_ to produce training sets, to which linear models were fit using each one of the imbalance statistics as a predictor. Each model was then used to predict *c*_1_ from the remaining trees as validation sets. Results of this analysis are summarized in table 1. There was no significant improvement in predictive accuracy from combining multiple imbalance statistics as predictors (data not shown). Overall, the performance of imbalance statistics for predicting *c*_1_ tended to be poor ($R^{2}<5\%$). Shao and Sokal’s *b*_2_ statistic was consistently the best among the imbalance statistics. Like Sackin’s index, *b*_2_ is based on the sum of path lengths from the *i*th tip to the root (*l_i_*, measured in the number of nodes), but normalizes these lengths by $2^{l_{i}}$ (Shao and Sokal 1990). The highest value of $R^{2}=65\%$ across imbalance statistics was obtained by applying *b*_2_ to trees with preferential mixing where *c*_1_ constrained to values less than or equal to 1. However, *b*_2_ was not robust to varying these conditions and the *R*^2^ values under different conditions were much lower (<30%; table 1). If *b*_2_ was used to train a linear model across the entire range of *c*_1_, the median *R*^2^ value was negative (−0.12%), indicating that the predictive value of the model was worse than a naive model. In contrast, a kernel support vector regression model with an *ϵ*-insensitive loss function (*ϵ*-SVR) trained on these same data consistently obtained *R*^2^ values above 68% for the entire range of *c*_1_; that is, without benefiting from any prior information about *c*_1_.

**Table 1.** Predicting Contact Rates from Tree Shape.

Mixing	Method	$c_{1}\leq 1$			$c_{1}\geq 1$		
		Median $R^{2}$	25%	75%	Median *R*^2^	25%	75%
Proportional	Kernel	*69.5*	*69.4*	*69.7*	*69.5*	*69.4*	*69.7*
(ρ = 0)	# cherries	1.6	0.33	2.2	1.2	0.1	1.5
	*I_S_*	7.5	5.6	9	1.8	0.41	2.4
	*I_C_*	6.3	4.1	8	1.8	0.49	2.4
	Shao-Sokal *b*_1_	1.4	0.43	2	0.94	0.17	1.5
	Shao-Sokal *b*_2_	18	16	20	3.8	2.5	4.5
	∑*f*	1.6	0.68	2	0.82	−0.16	1.2
	$\overset{¯}{f}$	1.3	0.32	1.5	0.62	−0.24	0.96
	${\overset{¯}{f}}_{10}$	2.7	1.2	3.4	2.3	0.97	3.1
Preferential	Kernel	*68.5*	*68.4*	*68.7*	*68.5*	*68.4*	*68.7*
(ρ=0.9)	# cherries	1.7	0.74	2.3	−0.25	−0.76	0.01
	*I_S_*	12	9.3	13	3.1	1.6	4.2
	*I_C_*	6.6	4.5	8.5	1.7	0.33	2.5
	Shao-Sokal *b*_1_	3.2	1.7	4.2	0.38	−0.44	0.66
	Shao-Sokal *b*_2_	65	63	68	25	21	27
	∑*f*	3.5	2.3	4.1	0.5	−0.17	0.81
	$\overset{¯}{f}$	3.5	2.3	4.8	1	0.25	1.5
	${\overset{¯}{f}}_{10}$	−0.34	−0.89	−0.03	−0.35	−1	−0.05

Note.—For each imbalance statistic, a linear model was fit to random subsets of the trees and used to predict *c*_1_ (the contact rate of group 1) for the remaining trees. Each tree related 1,000 tips. The training set of trees was truncated to *c*_1_ greater or less than 1 (*n* = 200), as none of the imbalance statistics was able to differentiate between these scenarios (fig. 1*A* and supplementary fig. S3, Supplementary Material online). Similarly, random subsets of trees for all values of *c*_1_ (*n* = 350) were used to train a *ϵ*-SVR. Model performance was quantified using *R*^2^, reported here as a percentage. Note that the kernel method was applied to the entire range of *c*_1_.

### Approximate Bayesian Computation

One of the limitations of regression-based methods for inferring epidemiological model parameters from tree shapes is that it requires training data where these parameters are known (supervised learning). In the case of infectious disease epidemiology, it can be very difficult to obtain reliable estimates of the epidemiological parameters such as the total size of the infected population. It would be far more useful to be able to infer such parameters directly from the shape of a phylogenetic tree without any prior information. Here, I evaluate the use of ABC to fit an epidemiological model to the shape of a tree. ABC avoids having to calculate the exact likelihood of a model; instead, the model is used to simulate data sets, which are then compared with the observed data (Sunnåker et al. 2013). This comparison requires a distance measure that will map pairs of data sets to the real number line. For example, imbalance statistics could potentially be used for this purpose. Based on the poor performance of these statistics as reported in the preceding section, however, this analysis focuses specifically on using the tree shape kernel as a distance measure for parameter estimation with ABC.

The kernel function was employed to sample parameters from the posterior distribution in the framework of MCMC for ABC (ABC–MCMC; Marjoram et al. 2003). To reduce stochastic variation in kernel scores for a given model state, the kernel score was averaged over replicate tree simulations at each MCMC step. Otherwise an unusually high kernel score could occur by chance, effectively trapping the chain sample at that point of parameter space until the next extreme score, resulting in less efficient convergence of chain samples to the approximated posterior distribution. Although increasing the number of tree replicates improves convergence (Sisson and Fan 2011), it carries a linear computational cost and diminishing returns with the number of replicates. The results presented here were obtained from chains with *n* = 10 replicate simulations. Chain sampling was also improved by the use of an exponential weighting kernel for calculating the acceptance probability of a proposal (Ratmann et al. 2012).

To evaluate the performance of ABC–MCMC, I generated trees under the BDSIR model (Kühnert et al. 2014) with varying numbers of dated tips under three scenarios (supplementary table S1, Supplementary Material online). Parameter settings for the BDSIR simulations were derived from previous work (Popinga A, Vaughan T, Stadler T, Drummond A, unpublished data, http://arxiv.org/abs/1407.1792v1, last accessed June 15, 2015) and examples in the BEAST2 distribution. The BDSIR model stipulates that virus lineages are “born” upon the transmission of the virus between infected and susceptible individuals at a rate *β.* Lineages die at a rate *γ* corresponding to the removal of an infected individual from the population because of recovery, behavioral change, or mortality. In addition, lineages terminate at tips of the tree when an infection is sampled from the population at a rate ϕ. Thus, the BDSIR model assumes that no subsequent transmissions originate from sampled infections. Each tree was used to generate a sequence alignment by simulation, which were then processed by two different approaches. First, I used the phylodynamic kernel-ABC framework to fit the BDSIR model to trees that were reconstructed from these alignments by maximum likelihood and rooted under a strict molecular clock. Second, I used the phylodynamics module in BEAST2 (Bouckaert et al. 2014) to fit the BDSIR model using Bayesian MCMC sampling with exact likelihoods.

**Fig. 2.**
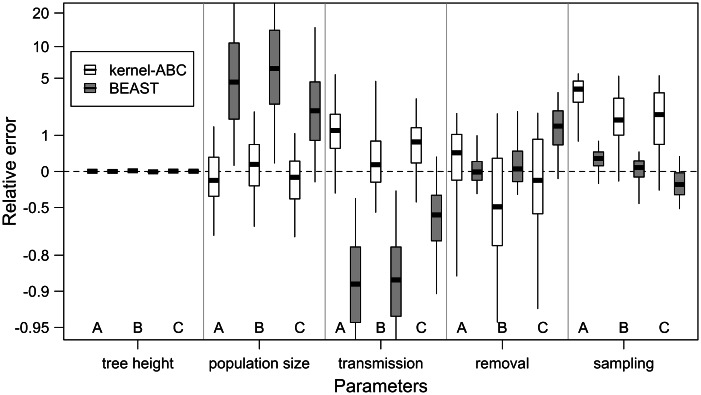
Comparison of relative errors for posterior estimates of BDSIR model parameters obtained by kernel-ABC and BEAST2. Each box-and-whisker plot summarizes the parameter distribution sampled from the posterior density. The relative error was calculated as $(\hat{x}-x)/x$ where $\hat{x}$ is the estimated value and *x* is the true value; hence, a relative error of −0.9 corresponds to a 10-fold underestimate. These quantities were offset by 1 for rendering on a log-transformed scale. The vertical span of boxes corresponds to the interquartile range and the whiskers extend to the empirical 95% credible intervals. Population size corresponds to the total number of susceptible, infected and removed individuals. Detailed results are provided in supplementary table S1, Supplementary Material online.

Figure 2 summarizes the posterior distributions of the BDSIR model parameters obtained using kernel-ABC and BEAST2 methods. Chain samples from BEAST2 consistently overestimated the total population size *N* by roughly a factor of 5. I verified that these posterior estimates from BEAST2 were substantially displaced from the prior distributions, indicating that the simulated data were sufficiently informative. In contrast, kernel-ABC was remarkably successful at recovering the actual values of *N* across all three scenarios within ≈20% of the actual value. Kernel-ABC was also able to recover the actual values of the transmission rate (*β*) across scenarios, whereas BEAST2 tended to underestimate *β* by a factor of 10 (fig. 2). These parameters are associated with lineage birth rates in the BDSIR model. Posterior samples of *N* and *β* from kernel-ABC exhibited an inverse proportional relationship, resulting in a significant negative correlation (Spearman’s $\rho <-0.6,\;P<10^{-12}$). Thus, the products of *β* and *N* were more comparable between BEAST2 and kernel-ABC. In contrast, BEAST2 provided more accurate estimates of the lineage death parameters *γ* and ϕ (mortality and sampling rates) with smaller credible intervals (fig. 2). Both approaches were successful in reconstructing the height of the tree (*T*), which approximates the origin of the epidemic, from sequence variation. In the case of kernel-ABC, a root-to-tip method (Rambaut 2000) was applied to a maximum-likelihood reconstruction of the phylogeny to estimate *T.* Similar results were obtained by fixing the tree in BEAST2 to the maximum-likelihood phylogeny by disabling all MCMC operators associated with modifying the tree topology (results not shown).

A comparison of computing times from the kernel-ABC and BEAST2 experiments with the BDSIR model is provided in supplementary table S2, Supplementary Material online. Overall, the kernel-ABC algorithm ran about 3–4 orders of magnitude slower than BEAST2. This is not surprising because BEAST2 can reuse much of its likelihood calculations on trees that are only slightly modified between steps in the chain sample. In contrast, an ABC–MCMC must constantly regenerate trees by simulation and compute kernel scores de novo at every step of the chain. Computing the normalized kernel score required about 1.5 s for trees with 1,000 tips on a desktop computer running an Intel Xeon E5-1650v2 processor. As predicted, this step had a time complexity of $O(n^{2})$ where *n* was the number of tips. Although kernel-ABC was at a clear disadvantage with respect to computational speed, results in supplementary table S2, Supplementary Material online suggest that kernel-ABC may require far fewer steps to obtain effective sample sizes comparable to long chains generated in BEAST2.

**Fig. 3.**
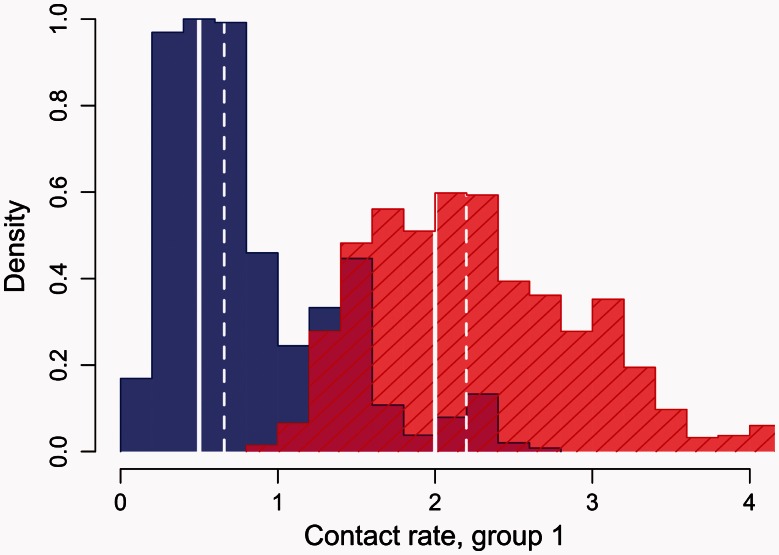
Histograms summarizing the distribution of *c*_1_ (contact rate, group 1) estimated by a kernel-ABC analysis on trees simulated with $c_{1}=0.5$ (blue) and $c_{1}=2.0$ (red, hatched). The true parameter settings and median estimates are indicated by solid and dashed white vertical lines, respectively. Both runs were initialized at $c_{1}=1$. The simulated annealing tolerance parameter was set to decay from 0.005 to 0.002.

Finally, I used the kernel-ABC method to fit the differential risk model (Jacquez et al. 1988) to the shapes of phylogenies, which were reconstructed from sequences that were generated with transmission trees simulated under this model (for $c_{1}=0.5$ and $c_{1}=2.0$). This is the same model that was used to generate the trees in our initial assessment of imbalance statistics and the kernel method, as summarized in figure 1. Figure 3 demonstrates that kernel-ABC was able to recover the actual values of *c*_1_. Simultaneously, the model parameters *N*, *β* and *γ* were also estimated by kernel-ABC, which had the actual values of 3,000, 0.01, and $\gamma =1.92\times 10^{-3}$/week, respectively. As *c*_2_ is confounded for varying *β* and *c*_1_ (eq. 1), this parameter was fixed at 1.0. I observed a significant correlation between values of *N* and *γ* in the chain samples (Spearman’s $\rho =0.53,\;P=1.5\times 10^{-8}$). Even so, these analyses were able to obtain reasonable estimates of these parameters. The respective medians and interquartile ranges for *N* were 4,620 (3,446, 6,330) and 2,628 (1,735, 3,676) for the $c_{1}=0.5$ and $c_{1}=2.0$ scenarios, respectively. For *β*, these were 0.014 (0.012, 0.017) and 0.0092 (0.0074, 0.012); for *γ*, these were 1.8 (0.9, 2.2)$\times 10^{-3}$ and 1.7 (1.0, 2.1)$\times 10^{-3}$. These results demonstrate the inherent versatility of the kernel-ABC method; to date, there is no published software that can fit this differential risk model using conventional Bayesian methods based on exact likelihoods.

### Application to HIV Data

**Fig. 4.**
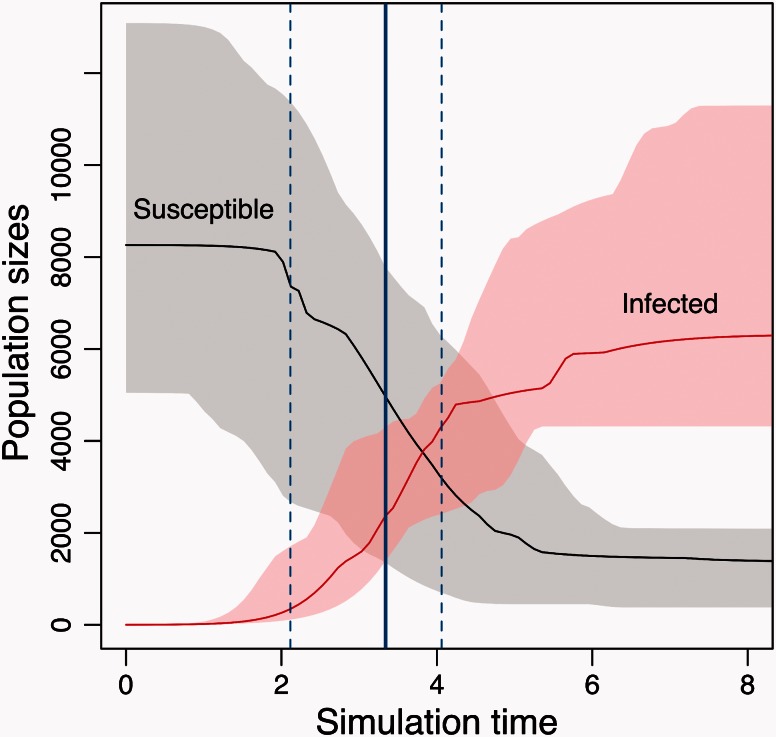
Reconstructed dynamics of a recent outbreak of HIV CRF07_BC in China. Numbers of susceptible (gray) and infected (red) individuals over time were obtained by forward-time simulation in MASTER under the birth–death SIR model, using parameter values sampled by kernel-ABC. Shaded areas indicate the interquartile regions of the respective counts. Note that the infected counts include removed individuals from simulated populations. A solid vertical line indicates the median tree height that approximates the date of the most recent sample in the data set (2010). Dashed lines indicate the interquartile range in tree heights. Simulation time zero corresponds to the estimated origin of the epidemic (1993–1994).

The preceding section demonstrated that combining ABC–MCMC with a tree shape kernel provided accurate estimates of “birth” parameters of the BDSIR model from data that were simulated under the same model. The next step was to observe how this method responded to real-world data. Because the BDSIR model assumes a constant rate of sampling from an epidemic, it may be more appropriate to apply this model to a recent epidemic that spans a period of time where conventional genetic sequencing would have been readily available. For this reason, I selected the HIV CRF07_BC epidemic in China to evaluate this model. First identified in 1997 (Piyasirisilp et al. 2000), this circulating recombinant form (CRF) is the predominant HIV subtype among injection drug users in northwestern China (Xinjiang). Using a coalescent molecular clock analysis, Tee et al. (2008) estimated the origin of the CRF07_BC epidemic to be 1993.3 (95% highest posterior density [HPD]: 1991.2–1995.2). I obtained a similar estimate (1993.7) using a root-to-tip method (Rambaut 2000) to analyze a larger sample of *n* = 314 CRF07_BC sequences from China. Using kernel-ABC to fit the BDSIR model to this phylogeny, I obtained median estimates of *N* = 8,251 (95% CI 2,381–23,511) and β=3.86 (0.83–23.9) $\times 10^{-4}$. Inferred trajectories of the numbers of susceptible and infected individuals over time based on the sampled parameter values are summarized in figure 4. Using sampled tree heights to calibrate the simulation time units in MASTER to years, these trajectories indicated that epidemic was near the midpoint of its exponential growth phase by the most recent collection date of these data (2010), with a predicted maximum prevalence of about 6,620 (interquartile range 4,301–10,982).

Results in the preceding section indicated that BEAST2 should provide more reliable estimates for “death” parameters of the BDSIR model, namely the rates of removal (*γ*) and sampling (ϕ). The CRF07_BC data were analyzed using the serial BDSIR model implemented in the phylodynamics module in BEAST2. A relaxed uncorrelated lognormal clock model conferred a significantly improved fit to the data relative to a strict clock ($\log_{10}$ Bayes factor =93.0). This model obtained median estimates of γ=1.2 (95% HPD: 0.6–2.0) and ϕ=7.1 (95% HPD: 1.5−19) $\times 10^{-3}$, respectively. Consistent with the model validation results in the preceding section, BEAST2 obtained a roughly 5-fold higher median estimate of *N* = 48,151 (95% HPD: $5969-1.91\times 10^{5}$) and roughly 10-fold lower median estimate of β=4.81 (95% HPD: 0.82−14.3) $\times 10^{-5}$ in comparison to the kernel-ABC results.

## Discussion

Here, I have presented results demonstrating that a kernel function can provide a more informative quantification of phylogenetic tree shapes than summary statistics such as tree imbalance (fig. 1). This confers an enormous advantage to kernel methods when classifying tree shapes generated under varying epidemiological scenarios, even when varying aspects of tree shape to which imbalance statistics are the most attuned (specifically, variation in branching rates; table 1). Incorporating the tree shape kernel into an ABC framework provided more accurate estimates of the lineage birth parameters of the BDSIR model (population size *N* and transmission rate *β*) than obtained using BEAST2. On the other hand, BEAST2 outperformed the kernel-ABC method in recovering BDSIR parameters associated with lineage death, namely the rates of removal (*γ*) and sampling (ϕ). This outcome implies that the present formulation of the tree shape kernel function is more sensitive to the internal structure of the tree, and less sensitive to the distribution of tips over time. Kernel-ABC seems to benefit from preserving the branching-order of the tree, however, as opposed to methods like Kingman’s coalescent that summarize tree shape as a vector of coalescence times. Finally, I returned to the differential risk model used to evaluate the tree imbalance statistics, and showed that kernel-ABC could be used to fit this model to tree shapes.

This is not the first study to adapt ABC for phylodynamic inference. In a ground-breaking paper, Ratmann et al. (2012) implemented a similar ABC–MCMC method to fit a highly sophisticated model to both an influenza A virus (IAV) serotype H3N2 phylogeny and the surveillance data of weekly incidence in the Netherlands from 1994 to 2009. The distance metric for fitting this model was a function of nine summary measures, of which four were based on surveillance data (such as the mean number of case reports per season) and the remaining five were derived from sequence variation and the phylogeny (such as the time to the most recent common ancestor of all sequences from the same season). They concluded that key phylodynamic parameters could be estimated by ABC, but that the summary statistics used would most likely have to be customized from one study to another, as determined by the type of data available and characteristics of the virus. The key difference between their study and the current work is that I am using only a kernel function as a distance measure, which operates exclusively on the shapes of phylogenetic trees. As it does not incorporate surveillance data, this approach may result in a loss of statistical power; however, it also makes the kernel-ABC approach more generalizable. Furthermore, the kernel function can be extended to incorporate other types of information (discussed below). Other studies have applied tree shape statistics to reject null models of virus evolution and epidemiology, but without the full ABC treatment. For example, Koelle et al. (2006) used Colless’ index (*I_C_*) to compare trees simulated from a complex map of genotypes to antigenic space and disease transmission model of IAV to an actual phylogeny. Leventhal et al. (2012) used Sackin’s index (*I_S_*) to quantify deviations in tree imbalance from null distributions generated from epidemic simulations on randomly generated contact networks of individuals. More recently, Colijn and Gardy (2014) used a number of tree shape statistics to develop machine learning classifiers for phylogenies representing simulated outbreaks of tuberculosis, which were driven by “superspreader” or chain-like dynamics.

A key advantage of a simulation-based ABC framework for phylodynamic inference is that it can be highly versatile. It eliminates the requirement of Bayesian MCMC for a closed form solution for the likelihood of a model, or avoids cost of evaluating a complex likelihood function (Sunnåker et al. 2013). Thus, any model that can generate transmission trees can conceivably be used to estimate parameters from a virus phylogeny. Historically, it was only practical to simulate trees from a limited number of models, such as Yule’s model of speciation (Yule 1925), Kingman’s coalescent (Kingman 1982), or the ancestral selection graph (Krone and Neuhauser 1997). This range was significantly expanded by the seminal work by Volz (2012) on deriving coalescent processes from nonlinear epidemiological models. Such advances, coupled with kernel-ABC, represent an important opportunity to relax some of the core assumptions of phylodynamic inference (Frost et al. 2014). For example, I have demonstrated the use of a kernel-ABC method to fit a differential risk model to tree shapes (fig. 3), where the host population is structured by assortative groups with different contact rates. This provides an example of using kernel-ABC to fit a model that is not yet possible to fit with conventional Bayesian methods that require exact likelihoods.

A pervasive assumption in phylodynamics is that the internal nodes of the phylogeny correspond to transmission events between hosts over time. This is almost certainly unrealistic for virus populations such as HIV, where the effective population size within hosts can be on the same scale as the number of viral generations between transmission events (Maddison and Knowles 2006). A branching point in a virus sequence phylogeny represents the inferred common ancestor of two virus lineages. When those lineages were sampled from different infections, one or more transmissions between hosts must have occurred at any point(s) along either branch. The time difference between the common ancestor and a transmission event (in virus generations) scales roughly with the effective population size (*N_e_*) within the host where the lineages coalesce (Romero-Severson et al. 2014). *N_e_* is close to 1 at the start of infection due to a transmission bottleneck, followed by exponential growth to $N_{e}\approx 10^{3}-10^{5}$ (Kouyos et al. 2006). The node heights of an HIV phylogeny are therefore most likely further back in time than the nodes of the transmission tree. In addition, the discordance between the virus phylogeny and transmission tree may be exacerbated by incomplete lineage sorting (Maddison and Knowles 2006), where the order of coalescent events does not correspond to the order of transmission events from the same source infection. Finally, there is growing evidence of a selective bias for new infections to be founded by archived HIV variants from the acute stage of the source infection (known as the “store-and-retrieve” hypothesis; Vrancken et al. 2014). This process may also bias the node heights of an HIV phylogeny further back in time relative to the implied transmission events. Therefore, incorporating within-host evolutionary processes into increasingly complex phylodynamic models may be of critical importance. The kernel-ABC framework makes it feasible to incorporate within-host processes into phylodynamic inference, as the only prerequisite for attempting to fit a model to a phylogeny is the ability to simulate trees.

Further investigation of the kernel-ABC approach will incorporate labeled trees into this framework. Indeed, the tree kernel from which the kernel function was derived was developed for comparing the parse trees of sentences in which the labels correspond to words (Collins and Duffy 2002). In the context of phylodynamics, these labels may correspond to risk factors or stages of infection, which may confer greater power for estimating group-specific parameters. In addition, kernel-ABC can be extended to infer the structure of the host population from tree shapes by simulating transmission trees within contact networks (Leventhal et al. 2012). A key challenge in this future direction will be to implement or adapt an epidemic network simulation algorithm that will be sufficiently fast to use for ABC–MCMC. Finally, expanding the spectrum of generative models for trees in kernel-ABC should include combining the evolution of viruses within hosts with epidemiological processes among hosts. Taking this direction will bring the field closer to the original concept of phylodynamics, in which the shapes of virus trees are determined by processes operating both within and among hosts (Grenfell et al. 2004).

## Materials and Methods

### Simulating Tree Imbalance

Phylogenetic trees were generated using a Python module implemented by E. Volz (colgem2, https://code.google.com/p/colgem/, last accessed June 15, 2015) in which a broad range of epidemic models—expressed as a system of ordinary differential equations that can be solved by numerical integration using SciPy function “odeint” (http://www.scipy.org, last accessed June 15, 2015)—can be used to determine the coalescent process (Frost and Volz 2013). As tree imbalance statistics were designed to detect variation in branching rates along lineages, I chose an epidemic model in which the host population was structured into groups with different risks of transmission for the basis of comparison (Jacquez et al. 1988). In brief, the conventional susceptible–infected (SI) compartmental model is partitioned into nonoverlapping subpopulations whose dynamics are described by the following set of differential equations:

(1) dSidt=Λi−Si(∑jβcipijIjSj+Ij−μ),dIidt=Si(∑jβcipijIjSj+Ij)−Ii(μ+γ),

where Λ*_i_* is the constant input rate of new susceptible individuals in group *i* (*S_i_*). Infection causes an elevated rate of mortality by an amount *γ* above the baseline rate *μ. β* is the per-contact transmission rate. Individuals in the *i*th group experience *c_i_* contacts per unit time, with a fraction *p_ij_* of these contacts reserved for individuals in group *j.* Here, I will use the “preferred mixing” formulation introduced by Jacquez et al. (1988) to define *p_ij_* in terms of homophily, where a fraction *ρ* of an individual’s contacts are reserved for peers from their same group:

$$p_{ij}=\text{\&}\{\begin{array}{cc} \rho +(1-\rho )\frac{m_{i}}{\sum_{k}m_{k}} & \text{if}j=i\\ (1-\rho )\frac{m_{j}}{\sum_{k}m_{k}} & \text{if}j\neq i \end{array}\text{\&},$$

where $m_{k}=c_{k}(S_{k}+I_{k})$. Following Frost and Volz (2013), I have made the simplifying assumption that *ρ* does not vary among groups. In this study, the number of groups was limited to 2 to facilitate interpretation. The numerical solution of this system was used to simulate a coalescent process using a customized version of colgem2 that can generate replicate trees from a single function call.

Epidemics were initialized with a susceptible population size $N=10^{4}-1$ with a single infected individual in risk group 1. The susceptible population was partitioned into Np−1 and N(1−p) individuals in risk groups 1 and 2, respectively, where *p* is a parameter between 0 and 1. For these simulations, *p* was fixed to a value of 0.5. To facilitate comparison with previous work, the differential risk model parameter values were set to those in Frost and Volz (2013); specifically: β=0.01 transmissions per contact; $\Lambda_{i}=S_{i}(0)/3,640$; μ=1/3,640; γ=1/520; and *ρ* = 0 for proportional mixing and ρ=0.9 for preferential mixing. Contact rates were set to $c_{2}=1$ for risk group 2 and varied for risk group 1 ($c_{1}\in \{0.125,0.25,0.5,1,2,4,8\}$). All rates were scaled to weeks. In total, 100 replicate coalescent trees each relating a sample of $n=10^{3}$ individuals were simulated for each setting of *c*_1_. Python scripts for generating these simulations are available at http://bioinfo.cfenet.ubc.ca/pub/kernel-abc (last accessed June 15, 2015).

### Quantifying Tree Shape

First, I applied a variety of conventional tree imbalance statistics to quantify the shapes of the trees simulated under the differential risk model. The number of “cherries” is calculated by the sum of internal nodes with exactly two terminal branch descendants (McKenzie and Steel 2000). Sackin’s index (*I_S_*) is defined as the total path length (depth) from a tip to the root as measured by the number of intervening nodes (Sackin 1972):

$$I_{S}=\sum_{j=1}^{n}\text{Depth}(j),$$

where *j* indexes over the tips of the tree. This index can be normalized for the size of the tree using the expected depth of tips under a Yule process (Blum and François 2005):

I^S=(IS−I¯S)/I¯S,whereI¯S=2n∑j=2n1j.

In addition, normalized Colless’ indices (Colless 1982; Kirkpatrick and Slatkin 1993), Shao and Sokal’s *B*_1_ and *B*_2_ statistics, and Fusco and Cronk’s imbalance measures (Purvis and Agapow 2002) were calculated for all trees. As outcomes did not vary qualitatively among imbalance measures, the results section focuses on Sackin’s index as being representative of this approach to quantifying tree shapes, with other results summarized in table 1 and supplementary figure S3, Supplementary Material online.

Additionally, I employed a kernel function to compare pairs of trees with respect to their shapes. This kernel function was previously developed for a comparative study of human and zoonotic RNA virus phylogenies (Poon et al. 2013) as an extension of the parse tree kernel proposed by Collins and Duffy (2002), for which an efficient algorithm was developed by Moschitti (2006). Briefly, the kernel operates by iterating over all pairings of the *i*th internal node in tree *T*_1_ and the *j*th internal node in tree *T*_2_ (denoted by $n_{1}^{i}$ and $n_{2}^{j}$, respectively) to identify the largest common subset tree rooted at these nodes. A subset tree is a contiguous structure nested within a tree that does not necessarily extend to the tips. Computing the inner product of this feature set results in a positive semidefinite kernel:

(2) k(T1,T2)=〈ϕ(T1),ϕ(T2)〉=∑i∑jΔ(n1i,n2j)Δ(n1i,n2j)=λkG(n1i,n2j)[1+Δ(l(n1i),l(n2j))][1+Δ(r(n1i),r(n2j))],

where *l*(*n*) and *r*(*n*) return the left and right nodes descending from node *n*, respectively, and *λ* is a constant decay factor to avoid the “diagonal dominance” problem by penalizing large subset trees (Collins and Duffy 2002). Equation (2) is adapted for phylogenetic or coalescent trees by weighting these features by their discordance in branch lengths using a Gaussian radial basis function (Poon et al. 2013). Specifically, the term $\Delta (n_{1}^{i},n_{2}^{j})$ was adjusted by a factor:

(3) $$k_{G}(n_{1}^{i},n_{2}^{j})=\exp\left(-\sigma^{-1}\left[{\left(\left|l(n_{1}^{i})|-|l(n_{2}^{j})\right|\right)}^{2}+{\left(\left|r(n_{i}^{i})|-|r(n_{2}^{j})\right|\right)}^{2}\right]\right),$$

where |l(n)| and |r(n)| extract the branch length between node *n* and its left and right descendant nodes, respectively, and $\sigma^{2}$ is the Gaussian variance parameter. Thus, *k_G_* assumes its maximum value of 1 when $l(n_{1}^{i})=l(n_{2}^{j})$ and $r(n_{1}^{i})=r(n_{2}^{j})$, and decays to zero with increasing discordance in branch lengths. As σ→∞, *k_G_* approaches 1 for all subset trees and we recover the original tree parse kernel of Collins and Duffy (2002).

All trees were ladderized by rotating branches around internal nodes such that the branch with the greater number of descendants was always located on the same side (left or right). As the kernel function makes a distinction between the left and right child branches of each node in a tree, this step confers greater consistency in comparing trees with similar topologies. In addition, the branch lengths in all trees were rescaled by a global factor such that the mean branch length was 1.0 units, to facilitate the comparison of trees of different overall lengths.

The kernel score from each pairwise comparison was normalized to facilitate comparisons between trees of different sizes (number of tips) using the method proposed by Collins and Duffy:

(4) $$k\prime (T_{1},T_{2})=\frac{k(T_{1},T_{2})}{\sqrt{k(T_{1},T_{1})k(T_{2},T_{2})}}.$$

The resulting matrix of kernel scores has been shown to be positive semidefinite (Poon et al. 2013). The kernel function was implemented in Python with the BioPython “Phylo” library (Talevich et al. 2012).

### Model Prediction

For each imbalance statistic, a linear model was trained on random subsets of the simulated trees, stratified by the target parameter. The model was then used to predict the parameter from imbalance statistics calculated from the remaining trees. For the kernel method, we generated the kernel matrix for the training set and then used this matrix to train a *ϵ*-SVR (Drucker et al. 1997) using the R package “kernlab” (Karatzoglou et al. 2004). Prediction accuracy was quantified using the *R*^2^ statistic, which can be loosely interpreted as the proportion of the variation explained by the model:

$$R^{2}=1-\frac{\sum_{i}{\left(x_{i}-\overset{¯}{x}\right)}^{2}}{\sum_{i}{\left(x_{i}-\hat{x}\right)}^{2}},$$

where $\overset{¯}{x}$ is the empirical mean of the variable and $\hat{x}$ is its predicted value.

### Approximate Bayesian Computation

To estimate model parameters from a single (reference) tree, I used the normalized kernel score (eq. 4) to quantify the similarity between this reference and one or more trees simulated from the model under a given set of parameters (*θ*). This approach belongs to a broad category of techniques known as ABC, which obviates the need to calculate exact likelihoods in fitting complex models. Specifically, I employed a type of ABC inference known as ABC–MCMC, which employs the likelihood-free method in the MCMC framework (Marjoram et al. 2003). First, a chain was seeded with arbitrary parameter values (*θ*) for the model. This chain state was evaluated by simulating *n* trees from the model given *θ*, using either forward-time (MASTER; Vaughan and Drummond 2013) or coalescent (rcolgem, http://colgem.r-forge.r-project.org, last accessed June 15, 2015) methods. To facilitate comparison to BEAST2, the analyses reported in this article used the MASTER (version 2.0.0) program included in the BEAST2 software package. To reduce the computational cost of simulating *n* trees, replicate trees were simulated in parallel using a customized version of rcolgem; MASTER did not support parallel execution. Branch lengths in each tree were normalized by the mean branch length to facilitate comparison between trees measured on different scales. Normalized kernel scores (k′) were calculated for the pairwise comparison of each simulated tree to the reference tree. To accelerate this step, I used the Python modules “multiprocessing” and “dill” to distribute kernel computation across multiple cores. The overall score for *θ*, denoted by $\hat{k}(\theta )$, was estimated by averaging k′ over *n* replicates. Note that $\hat{k}$ ranges from 0 to 1 because of the normalization in equation (4).

Chain sampling proceeded using a Metropolis–Hastings algorithm as follows. A new set of parameter values (θ′) were drawn at random from a truncated multivariate proposal distribution centered at *θ*, with a mixture of Gaussian and lognormal distributions with predefined minimum, maximum, and standard deviation for each parameter. All parameters were modified by the proposal (full-dimensional updating), although similar results were obtained using componentwise (Gibbs) updating. The probability of accepting the proposed values was determined by computing $\hat{k}(\theta \prime )$ as above, and comparing this value to $\hat{k}(\theta )$ by an exponential weighting kernel (Ratmann et al. 2012):

(5) $$\alpha =\min(1,\frac{\exp[-2(1-\hat{k}(\theta \prime ))/\tau ]q(\theta \prime |\theta )\pi (\theta \prime )}{\exp[-2(1-\hat{k}(\theta ))/\tau ]q(\theta |\theta \prime )\pi (\theta )}),$$

where *τ* is a tolerance parameter. The analyses presented in this article assumed a uniform prior distribution (π(θ)=C) and symmetric proposal density (q(θ′|θ)=q(θ|θ′)). To prevent the chain sample from becoming stuck in low-scoring regions of parameter space, which is a well-known problem in ABC-based inference (Sisson and Fan 2011), I used simulated annealing where *τ* was adjusted over time by an exponential decay function:

$$\tau (t)=\tau_{\text{min}}+(\tau_{0}-\tau_{\text{min}})\exp(-\lambda_{\tau }t),$$

where *τ*_0_ was the initial tolerance value, $\lambda_{\tau }$ is an exponential decay rate, and $\tau_{\text{min}}$ is the minimum tolerance.

### Method Validation

To validate the kernel-ABC method for phylodynamic inference, I used MASTER (Vaughan and Drummond 2013) to simulate trees under a BDSIR model. This model specifies three compartments corresponding to susceptible (S), infected (I), and removed (R) individuals, where removal may correspond to clearance and immunity from reinfection, mortality, or behavior modification (Kühnert et al. 2014). In addition, the BDSIR model assumes that sampling individuals from the epidemic also removes them from the pool of infected individuals. This is assumed to occur at a constant rate (ϕ) in proportion to the current number of infected individuals. Using the reaction kinetics notation employed by MASTER, the BDSIR model can be expressed as follows:

$$\begin{array}{l} S+I\overset{\beta }{\to }2I\\ I\overset{\gamma }{\to }R\\ I\overset{\phi }{\to }I_{\text{sample}}. \end{array}$$

Following previous work (Popinga A, Vaughan T, Stadler T, Drummond A, unpublished data, http://arxiv.org/abs/1407.1792v1, last accessed June 15, 2015), the rate parameters for removal and sampling were set to γ=0.3 and ϕ=0.15, respectively. The birth (transmission) rate was set to $\beta =10^{-3},\;3\times 10^{-4}$, and $10^{-4}$ for *N* = 1,000, 3,000, and 10^4^ susceptible individuals at time 0, respectively. It was necessary to reduce *β* with increasing *N* to prevent explosive growth at the initial phase of the epidemic, resulting in a “star”-like phylogeny with unresolvable internal nodes. Under these varying conditions, trees were generated with exactly 100, 300, and 1,000 tips, respectively, by specifying the “leaf count” postsimulation condition in MASTER (Vaughan and Drummond 2013).

I used the software package INDELIBLE (Fletcher and Yang 2009) to simulate sequence evolution along each tree under the M3 model of codon evolution. This model defines a transition–transversion bias parameter (κ=8.0) and a discretized gamma distribution of nonsynonymous–synonymous rate ratios (*ω*) with 50 categories and a mean of 0.5 and standard deviation of 0.015. Branch lengths in the simulated trees were rescaled so that codon substitution events were expected to occur in about 10% of branches in the tree per codon. These settings yielded alignments of sequences (300 codons in length) resembling rapidly evolving regions of an RNA virus genome. Although slower rates of evolution may be more generalizable, the objective was to provide idealized conditions for parameter estimation using either BEAST2 or the kernel-ABC method—in other words, to identify biases inherent to either framework rather than due to uncertainty in phylogenetic reconstruction. An assessment of the sensitivity of parameter estimation to lower rates of evolution is provided as supplementary figure S5, Supplementary Material online.

The simulated alignments were each imported into BEAUTI (version 2.1.3). Tip dates, corresponding to the locations of tips in the tree produced by the MASTER simulation, were extracted from sequence labels. As the nucleotide sequence data were simulated with a constant rate of evolution and no rate variation among sites, the BEAUTI interface was used to specify an HKY85 model of nucleotide evolution (Hasegawa et al. 1985) without rate variation, a strict clock, and a serial BDSIR tree prior to match the conditions under which the sequence alignments had been generated. Other prior distributions were left at their default settings. The resulting XML files were executed with two replicate chains each for 10^8^ steps using the serial BDSIR model within the phylodynamics module in the software package BEAST2 (version 2.1.3; Bouckaert et al. 2014). Memory allocations were increased from their default settings to 512 and 1,024 MB for starting and maximum levels, respectively. To process trees with 1,000 tips, the maximum memory allocation was increased to 2,048 MB. Gelman and Rubin’s convergence diagnostic and effective sample sizes were calculated using the R package “coda” (Plummer et al. 2006). Replicate posterior traces in BEAST2 were consistent with convergence based on Gelman and Rubin’s diagnostic for all three scenarios (potential scale reduction factor = 1). Estimates of total population size (*N*) were derived from the initial number of susceptible individuals (*S*_0_) plus one.

To reconstruct phylogenies from the simulated alignments for phylodynamic inference with the kernel-ABC method, I used RAxML (version 8.1.3 Stamatakis 2014) with the GTRCAT model of nucleotide evolution. The resulting maximum-likelihood tree was converted into a strictly bifurcating tree using the “rtt” function in the R package “ape” (Paradis et al. 2004). A modified version of the RootToTip function, which was implemented in the Path-O-Gen application within the BEAST package (version 1.8.0; Drummond and Rambaut 2007), was used to extrapolate the root of the tree from tip dates under a strict molecular clock. The resulting time-scaled trees were processed within the kernel-ABC framework as described above, using MASTER to simulate *n* = 10 trees under parameter values drawn from the proposal distribution. Because using forward time simulation to generate a tree with an exact number of tips can result in a large number of discarded trees, this requirement was relaxed to increase the efficiency of this step. Although the kernel function does not require trees to have the same numbers of tips, tree shape comparisons can be sensitive to differences in tree size, so terminal branches in the simulated trees were randomly pruned to the same number of tips. The kernel decay parameter was set to λ=0.35. The Gaussian radial basis function tolerance parameter was set to σ=2.0 based on previous work in which it was determined that kernel methods performed best with *σ* around this value (Poon et al. 2013). Similar but slightly less accurate results were obtained using σ=0.5, which was consistent with Poon et al. (2013). Simulated annealing parameters were set to $\tau_{0}=0.02,\;\tau_{\text{min}}=0.01$, and $\lambda_{\tau }=0.0025$ for fitting the BDSIR model. A log-normal proposal was used for *N* with σ=0.2; Gaussian proposals were used for all other parameters. ABC–MCMC chains were propagated for about 10,000 steps.

A similar procedure used to simulate alignments under the differential risk model (Jacquez et al. 1988), with the exception that trees were simulated by implementing equation (1) within the rcolgem coalescent framework. Model parameters were set to $N=S+I=3,000,\;\beta =0.01,\;c_{2}=1$, *p* = 0.5, ρ=0.9, μ=1/3,640, and γ=1/520. With the exception of *N*, these settings reiterated the conditions used to simulate trees for evaluating imbalance statistics and the kernel function (fig. 1). The contact rate of group 1 was set to $c_{1}=0.5$ and $c_{1}=2.0$ to generate two sets of alignments. The kernel-ABC settings for analyzing this model were $\lambda =0.3,\;\sigma =2.0,\;\tau_{0}=0.005,\;\tau_{\text{min}}=0.002$, and $\lambda_{\tau }=0.0025$. Note that more aggressive simulated annealing was used due to less variation in kernel scores among trees simulated under different parameterizations of the differential risk model using rcolgem.

### HIV Data Processing

To reconstruct a phylogeny from real-world data, I queried the Los Alamos National Laboratory (LANL) HIV Sequence database (http://www.hiv.lanl.gov/, last accessed June 15, 2015) to obtain all published HIV CRF 07BC *env* gp120 sequences isolated in China with known years of collection (range 1997–2010). These records were restricted to one record per patient (*n* = 314) using the LANL interface, by manual inspection, and by excluding identical sequences. I generated a multiple sequence alignment from these sequences using MUSCLE (version 3.8.31; Edgar 2004). The resulting alignment was manually edited in AliView (Larsson 2014) to exclude regions of gp120 with substantial indel variation, which tend to produce alignment errors due to a lack of homology among inserted sequences. A rooted and timed phylogeny was reconstructed from this alignment using the same procedure as applied to simulated sequence data (see preceding section). An SIR model was fit to this tree by running an ABC–MCMC chain sample under the same conditions as used in model validation. The first 100 steps was discarded as a burn-in period. The same alignment was also processed using the serial BDSIR method in BEAST2. I specified a TN93 model with rate variation modeled by a gamma distribution with four rate classes and an invariant rate category. Additionally, I evaluated both strict and relaxed (uncorrelated lognormal) molecular clock models. To reconstruct the dynamics of susceptible and infected populations, parameter values were extracted from every 100 steps of the kernel-ABC chain sample and used to simulate BDSIR model trajectories and trees using MASTER. The mean and standard deviation of the model trajectories were exported using the “moment” tag in the MASTER XML specification (Vaughan and Drummond 2013).

## Data Availability

Scripts, alignments, and phylogenies are available at http://github.com/ArtPoon/kamphir (last accessed June 15, 2015).

## Supplementary Material

Supplementary tables S1 and S2 and figures S1−S5 are available at *Molecular Biology and Evolution* online (http://www.mbe.oxfordjournals.org/).

Supplementary Data
