# Supplementary material for: Phylodynamic Inference with Kernel ABC and Its Application to HIV Epidemiology
Source: Mol Biol Evol. 2015 May 29;32(9):2483–95. doi: 10.1093/molbev/msv123 (PMC4540972; doi:10.1093/molbev/msv123)
Supplement: Supplementary Data [file supp_32_9_2483__index.html]

Phylodynamic Inference with Kernel ABC and Its Application to HIV Epidemiology — Phylodynamic Inference with Kernel ABC and Its Application to HIV Epidemiology — Supplementary Data 

# Phylodynamic Inference with Kernel ABC and Its Application to HIV Epidemiology

## Supplementary Data

files

- Supplementary Data - pdf file
