# Supplementary material for: Phylodynamic Inference with Kernel ABC and Its Application to HIV Epidemiology
Source: Mol Biol Evol. 2015 May 29;32(9):2483–95. doi: 10.1093/molbev/msv123 (PMC4540972; doi:10.1093/molbev/msv123)
Supplement: Supplementary Data [file supp_msv123_kernel-abc_rev3_suppl.pdf]

SUPPLEMENTARY FIGURES

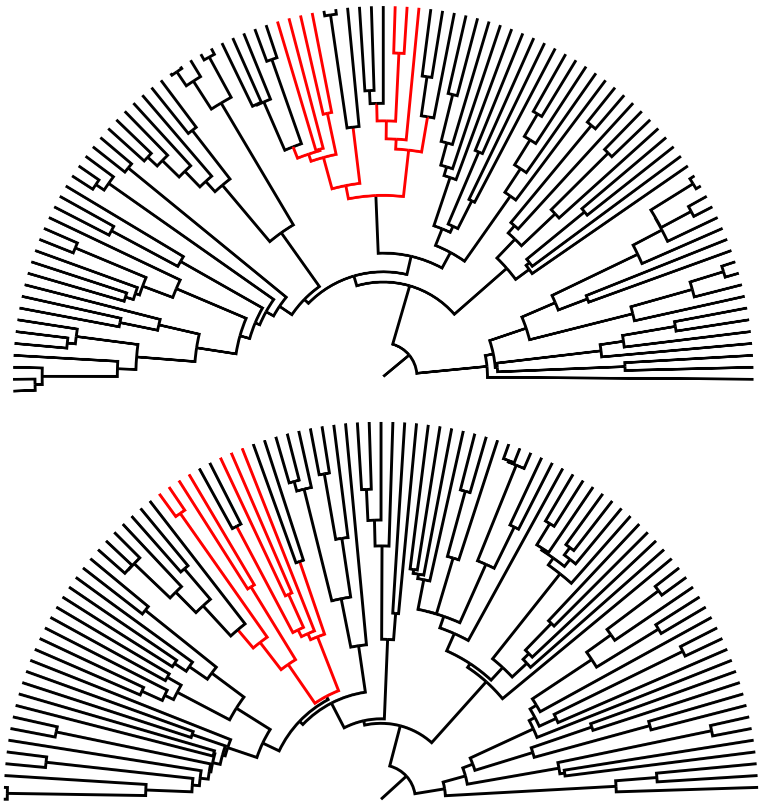

Figure S1: Matching subset trees between different phylogenies. The branches highlighted in red correspond to subset trees with the same branching order. Note that these subset trees do not have to extend from the internal node to all descendant terminal branches (tips), nor are they required to relate the same taxa in order to be counted as a matching feature towards the kernel score. Moreover, the discordance in branch lengths between these subset trees is accommodated by the kernel function via a Gaussian radial basis function (see Equations 2 and 4).

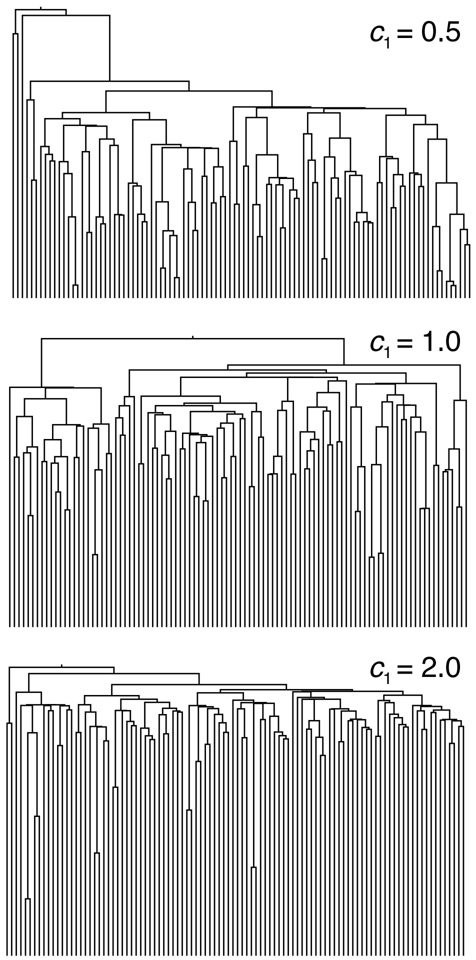

Figure S2: Effect of a single model parameter on tree shapes. These trees generated by simulating a coalescent process under a differential risk model with contact rates $c_2 = 1$ and c_1 varying from 0.5 to 2.0. Each tree relates $n = 100$ infected individuals sampled from the population; for clarity, this n is smaller than the sample size in the simulations used for model validation and inference ($n = 1000$). The lengths of branches leading to tips tend to be longer than branches closer to the root because the infected population is undergoing exponential growth.

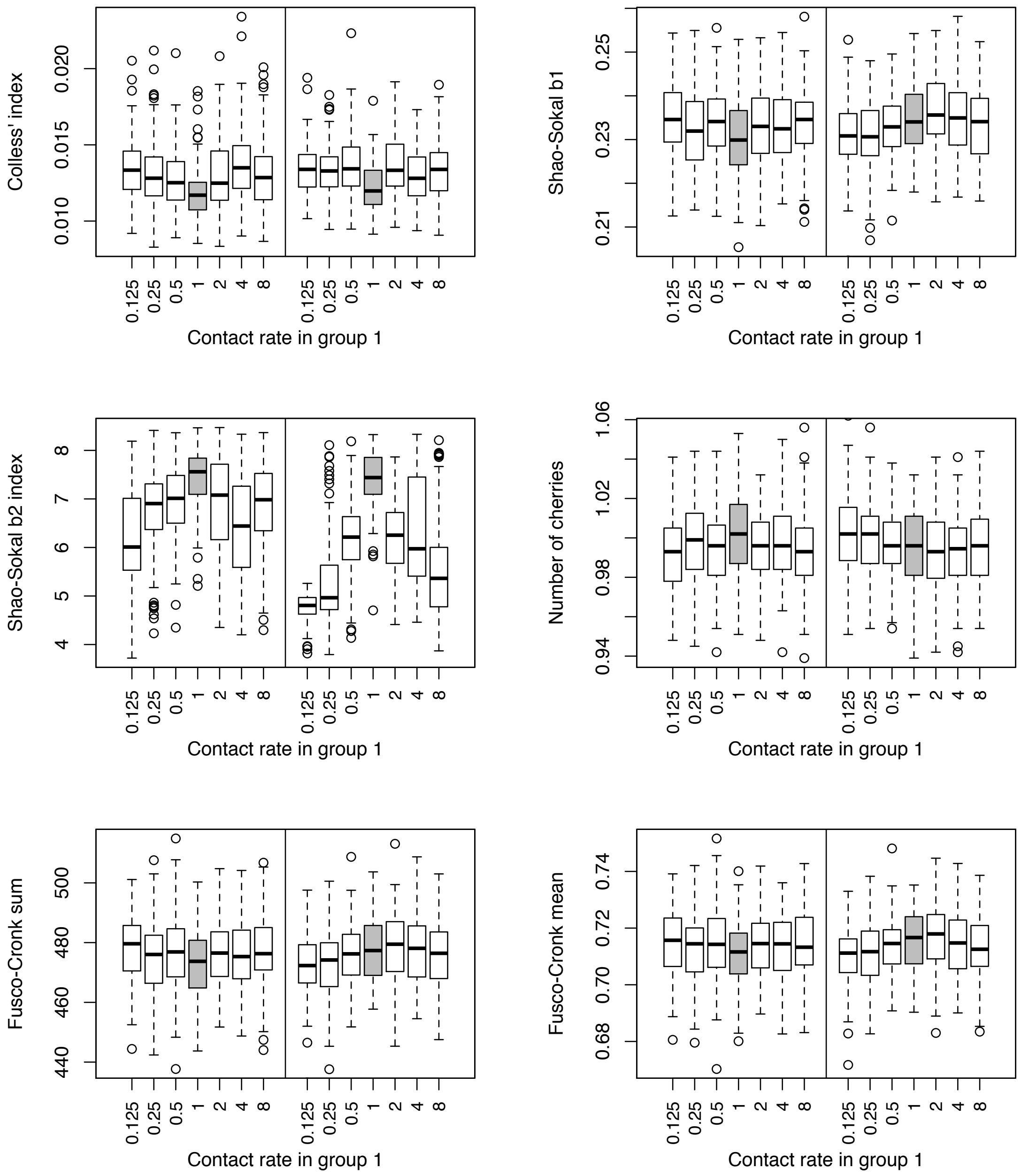

Figure S3: Response of other tree imbalance statistics to varying heterogeneity in contact rates. Results for Sackin's index (I_S) are presented as Figure 1A. Each set of box-and-whisker plots summarizes the distribution of the respective statistic (y-axis label) for 100 replicate trees simulated under different values of c_1 ($c_1 = c_2$ is shaded for reference). Results under proportional mixing ($\rho = 0$) are displayed on the left side, and preferential mixing ($\rho = 0.9$) on the right.

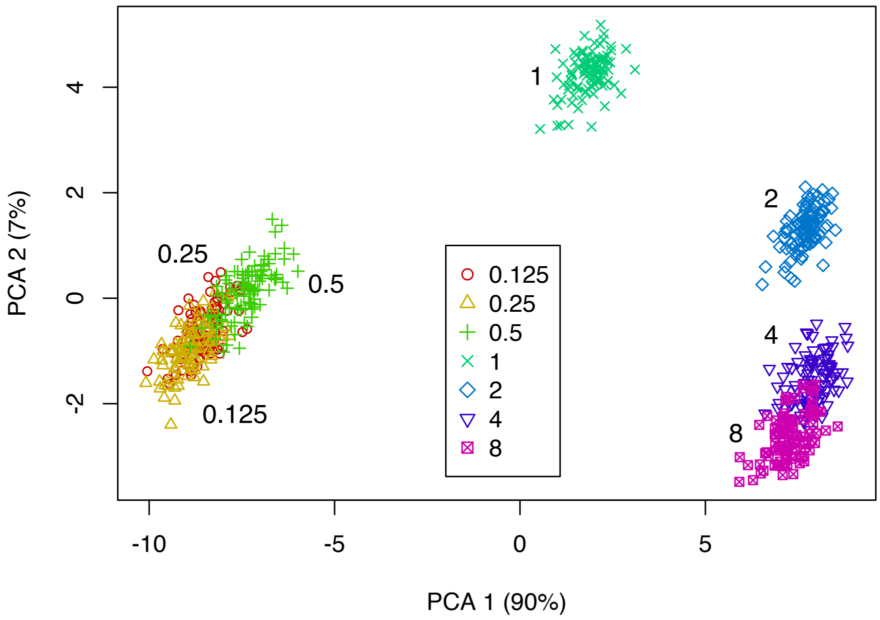

Figure S4: Projection of trees simulated under proportional mixing ($\rho = 0$) to kernel space. Results under preferential mixing ($\rho = 0.9$) are presented in Figure 1B. Sets of 100 replicate trees are each annotated in the plot with their corresponding c_1 values (also see colour/symbol legend in figure inset). The proportion of variation explained by the first two principal components, as estimated from the eigenvalues, is reported by the respective axis labels.

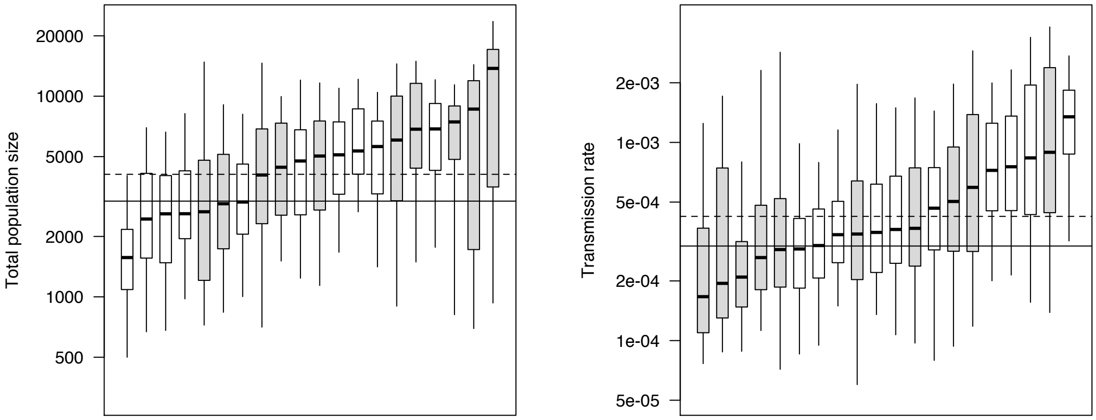

Figure S5: Replicate analyses on trees reconstructed from alignments of $n = 300$ sequences generated from a tree simulated under the BDSIR model with total population size $N = 3000$ (left) and transmission rate $\beta = 3 \times 10^{-4}$ (right), as indicated by solid horizontal lines. The median estimates are indicated with a bold line segment within a box representing the interquartile range. Whiskers extend from each box to indicate the empirical 95% credible intervals. Boxes are shaded to indicate alignments that were simulated under a two-fold lower rate of substitution. Dashed lines indicate the overall median parameter estimates.

SUPPLEMENTARY TABLES

Table S1: Performance of kernel-ABC and BEAST2 methods on simulated birth-death SIR (BD-SIR) trees. The median estimate for each parameter is reported alongside the empirical 95% confidence interval, or credible interval for BEAST2, in parentheses. Total population size N corresponded to the number of susceptible individuals at time 0 (S_0) plus one infected individual. Transmission rate was calculated from BEAST2 output as $\beta = R_0\gamma/S_0$. n = number of tips (sample size). T = time to the most recent common ancestor (origin of epidemic). Estimates of T for kernel-ABC inference were obtained from a maximum likelihood reconstruction of the phylogeny using a root-to-tip method.

**Scenario**	**Parameter**	**Actual**	**kernel-ABC**	**BEAST2**
A ($n = 100$)	N	1000	631 (228, 2940)	5586 (1121, 51282)
	$\beta \times 10^{-3}$	1.0	2.61 (0.26, 8.40)	0.11 (0.01, 0.6)
	γ	0.3	0.27 (0.02, 0.90)	0.30 (0.19, 0.60)
	ϕ	0.15	0.72 (0.14, 0.98)	0.19 (0.12, 0.27)
	T	19.68	19.75	20.96 (19.75, 24.35)
B ($n = 300$)	N	3000	3437 (1043, 9422)	21692 (3528, 231397)
	$\beta \times 10^{-3}$	0.3	0.34 (0.14, 1.7)	0.03 (0.004, 0.2)
	γ	0.3	0.15 (0.02, 0.91)	0.31 (0.19, 0.95)
	ϕ	0.15	0.4 (0.12, 0.94)	0.16 (0.08, 0.22)
	T	18.07	18.28	19.22 (17.98, 22.56)
C ($n = 1000$)	N	10000	8938 (2849, 20720)	32060 (8182, 158788)
	$\beta \times 10^{-3}$	0.1	0.18 (0.06, 0.40)	0.04 (0.01, 0.13)
	γ	0.3	0.25 (0.02, 0.92)	0.30 (0.19, 0.60)
	ϕ	0.15	0.45 (0.10, 0.94)	0.19 (0.12, 0.27)
	T	12.73	12.82	14.45 (13.12, 17.59)

Table S2: Comparison of computing times for kernel-ABC and BEAST2 experiments on simulated data. Effective sample sizes (ESS) were estimated from the posterior or kernel score traces using the *coda* package in *R*. Similar trends were observed when computing ESS from traces of model parameters. * indicates increased maximum memory allocation from 1024 to 2048 MB for improved performance; no further speed-up was attained with additional memory.

**Number of tips**	**Method**	**Time/1000 steps**	**ESS/1000 steps**	**ESS/h**
100	kernel-ABC	1.5h	27.5	18.3
	BEAST2	1.1s	0.056	183.3
	BEAST2 (fixed tree)	1.2s	0.032	99.4
300	kernel-ABC	6.8h	30.7	4.5
	BEAST2	1.8s	0.079	158.0
	BEAST2 (fixed tree)	1.6s	0.052	116.7
1000	kernel-ABC	15.1h	80.5	5.3
	BEAST2	29.7s*	0.014	1.7
	BEAST2 (fixed tree)	21.7s*	0.019	3.2
